# Astragalus Polysaccharides and Saponins Alleviate Liver Injury and Regulate Gut Microbiota in Alcohol Liver Disease Mice

**DOI:** 10.3390/foods10112688

**Published:** 2021-11-03

**Authors:** Jingxuan Zhou, Nanhai Zhang, Liang Zhao, Wei Wu, Liebing Zhang, Feng Zhou, Jingming Li

**Affiliations:** 1 Beijing Key Laboratory of Functional Food from Plant Resources, College of Food Science and Nutritional Engineering, China Agricultural University, 17 Tsinghua East Road, Beijing 100083, China; zjx888@cau.edu.cn (J.Z.); nanhaizhang@cau.edu.cn (N.Z.); lbzhang@cau.edu.cn (L.Z.); 2 Beijing Advance Innovation Center for Food Nutrition and Human Health, Beijing Engineering and Technology Research Center of Food Additives, Beijing Technology and Business University, 11 Fucheng Road, Beijing 100048, China; liangzhao@btbu.edu.cn; 3 College of Engineering, China Agricultural University, 17 Tsinghua East Road, Beijing 100083, China; wuweiyin@cau.edu.cn; 4 Center for Viticulture and Enology, College of Food Science and Nutritional Engineering, China Agricultural University, 17 Tsinghua East Road, Beijing 100083, China

**Keywords:** astragalus, alcoholic liver disease, polysaccharides, saponins, gut microbiota

## Abstract

Astragalus, a medical and edible plant in China, shows several bioactive properties. However, the role of astragalus in attenuating alcoholic liver disease (ALD) is less clear. The objective of this project is to investigate the improving effect of astragalus saponins (AS) and astragalus polysaccharides (AP), which are the two primary constituents in astragalus on hepatic injury induced by alcohol, and the potential mechanisms of action. Different doses of AS (50 and 100 mg/kg bw) and AP (300 and 600 mg/kg bw) were orally given to alcohol-treated mice for four weeks. The results demonstrated that both AP and AS could reverse the increase of the levels of TC, TG, FFA, and LDL-C in serum, and the decrease of serum HDL-C content, as well as the elevation of hepatic TC and TG levels induced by alcohol. The activities of AST, ALT, ALP, and γ-GT in ALD mice were raised after AP and AS supplementation. The antioxidant markers (SOD, CAT, GSH, and GSH-Px) were obviously augmented and the pro-inflammatory cytokines (TNF-α, IL-6 and IL-1β) and hepatic histological variations were alleviated by AP and AS, which was in line with the levels of oxidative stress-associated genes (*Keap1*, *Nfe2l2*, *Nqo1*, and *Hmox1*) and inflammation-associated genes (*Tlr4*, *Myd88* and *Nfkb1*). In addition, AS exerted a more efficient effect than AP and the results presented dose proportionality. Moreover, AS and AP could modulate the intestinal microbiota disturbance induced by alcohol. Overall, AS and AP administration could ameliorate lipid accumulation in the serum and liver, as well as hepatic function, oxidative stress, inflammatory response, and gut flora disorders in mice as a result of alcohol.

## 1. Introduction

Alcohol is very popular because of its particularly mellow flavor and rich variety. With the prevalence of wine culture, average alcohol consumption is increasing year by year, leading to an increasing risk of alcoholic liver disease (ALD) [1]. ALD could bring about a succession of pathological features of liver lesions, including hepatic steatosis, hepatitis, hepatic cirrhosis, hepatic fibrosis, and even hepatocellular cancer [2]. A study has reported that 90–100% of people addicted to alcohol could suffer from alcoholic fatty liver [3]. In addition, 5.9% of all deaths were caused by ALD and alcohol-related diseases, such as gastrointestinal diseases, central nervous system disorders, cardiomyopathy, malnutrition, etc. Evidence has shown that the mechanisms of ALD have been mainly attributed to oxidative stress and inflammation, as well as gut microbiota disorders [4]. Based on different mechanisms of action and targets, alcohol abstinence and some drugs such as corticosteroids, disulfiram, naltrexone, etc. have been used for managing ALD in clinical application [3]. Owing to the presence of toleration and adverse effects of drugs, the use of natural sources that possess properties of low toxicity and minimal side effects to alleviate ALD are popular at present.

As a legume plant, astragalus (Huangqi, *Astragalus membranaceus* Beg. var. *mongholicus* (Beg.)), one of the traditional Chinese medicines, has been applied commonly in China due to its biological activities, such as antioxidant, anti-inflammatory, hepatoprotective, immunomodulating, anti-cancer, and anti-photoaging properties [5,6,7,8]. Triterpene saponins and polysaccharides are believed to be the two main bioactive ingredients in Astragalus [9]. It has been revealed that astragalus saponins (AS) and astragalus polysaccharides (AP) have multiple bioactive properties. Liu et al. found that Astragaloside IV could inhibit the levels of pro-inflammatory cytokines through Toll-like receptor 4 (TLR4)/myeloid differentiation primary response gene 88 (MyD88)/nuclear factor kappa-B (NF-κB) signaling pathway, ultimately alleviating hepatic steatosis in mice induced by a high-fat diet [10]. Moreover, Astragaloside IV could inhibit the protein expression levels of protein tyrosine phosphatase 1B and sterol element regulatory binding protein-1c to block the triglyceride (TG) accumulation in HepG2 cells challenged on oleic acid [11]. It has been shown that Astragaloside IV exerts a beneficial function on mitigating diabetic nephropathy via suppressing oxidative stress, inflammatory response, epithelial-mesenchymal transition, as well as the Wnt/β-catenin signaling pathway [12]. Coincidentally, Astragaloside IV and AP could reverse the increase of blood lipids and decrease of hepatic function, as well as hepatic steatosis caused by a high-fat diet [10,13]. At the same time, Hong et al. further demonstrated that the changes of intestinal microbiota composition and function in mice resulting from a high-fat diet were ameliorated after AP supplementation. Dong et al. showed that AP could reduce the gene expression of inflammatory factors and chemokines by inhibiting the phosphorylation of NF-κB in lipopolysaccharide-stimulated porcine intestinal epithelial cells, and the alleviation of inflammatory response was verified in BALB/c mice after AP administration [14]. Studies have shown that AS and AP possess other biological activities, such as anti-tumor, anti-apoptosis, neuroprotection, and anti-Parkinson properties, as well as the improvement of insulin resistance [11,15,16,17], whereas the role of AS and AP in attenuating ALD remains poorly understood. In view of these bioactive properties above, we wondered whether AS and AP were potentially of benefit for the management of ALD.

Therefore, this work aims to evaluate the improving effects of saponins and polysaccharides extracted from astragalus produced in Hunyuan, Shanxi province on liver injury in alcohol-induced mice and its underlying mechanisms for inhibiting oxidative stress and anti-inflammation. Moreover, the influence of AS and AP on the gut microbiota of mice treated with alcohol was explored.

## 2. Materials and Methods

### 2.1. Materials and Chemicals

Astragalus was provided by Zeqingqi Industrial Development Co., Ltd. (Hunyuan, China). Macroporous resins (D101) were purchased from Sunresin New Materials Co., Ltd. (Xi’an, China). Edible alcohol was obtained from Henan Xinheyang Alcohol Co., Ltd. (Henan, China). The commercial kits of total cholesterol (TC), TG, high-density lipoprotein cholesterol (HDL-C), low-density lipoprotein cholesterol (LDL-C), alanine aminotransferase (ALT), aspartate aminotransferase (AST), and alkaline phosphatase (ALP) were purchased from Biosino Bio-Technology and Science Inc. (Beijing, China). The assay kits including γ-glutamyl transpeptidase (γ-GT), free fatty acid (FFA), malondialdehyde (MDA), catalase (CAT), superoxide dismutase (SOD), glutathione (GSH), glutathione peroxidase (GSH-Px), and enzyme-linked immunosorbent assay (ELISA) kits of tumor necrosis factor-α (TNF-α), interleukin-6 (IL-6), and interleukin-1β (IL-1β) were obtained from Beijing Sinouk Institute of Biological Technology (Beijing, China). TRIpure reagent was supplied by Aidlab Biotechnologies Co., Ltd. (Beijing, China). FastQuant RT Kit and SuperReal PreMix Plus with SYBR Green were purchased from Tiangen Biotech Co. Ltd. (Beijing, China). DNA extraction kit for soil was provided by Omega Bio-tek (Norcross, GA, USA). AxyPrep DNA Gel Extraction Kit was supplied from Axygen Biosciences (Union City, CA, USA). Other reagents, such as ethanol, calcium oxide, acetone, and NaOH, were all of analytical grade.

### 2.2. Preparation of Astragalus Saponins and Polysaccharides

The AS and AP were extracted by Xian Baichuan Biological Technology Co., Ltd. (Xi’an, China) according to their methods.

Astragalus was crushed, soaked in distilled water at a ratio of 1:6–7 (m/v), and extracted by thermal reflow for 2–3 h. The extracting solution was concentrated to a thick paste and 95% ethanol was added to achieve an ethanol concentration of 80%. After precipitation and filtration, the supernatant was concentrated to the thick paste again. The pH value of concentrated liquid was adjusted to 9 by NaOH solution and the alkaline liquor was loaded onto the pretreated macroporous resins (D101). Then the column was eluted with 10–15-fold the column volume of distilled water and 5–10-fold the column volume of 30% of ethanol, respectively. The elutes were collected after elution with 10 times the column volume of 80% of ethanol and evaporated. The concentrate was solved with a small amount of water, followed by rest for 24 h. After filtration, the residues were dried, namely AS. The content of AS was assayed to be 95% by using a vanillin colorimetric method with SpectraMax M2^e^ microplate reader (Molecular Devices, USA) in accordance with the procedure of Motz et al. [18].

The astragalus powder was mixed with calcium oxide solution at a ratio of 1:6–7 (m/v). The mixture was boiled for 1.5 h and then filtered. The sediments were extracted three times according to the method above. The pH value of blended filtrates was regulated to 6.5. Then the filtrate was concentrated and centrifuged at 2000× *g* for 10 min. The supernatant was mixed with 95% ethanol at a volume ratio of 1:3 to form the precipitate. After filtration, the residues were washed with acetone and then vacuum freeze-dried at −20 °C, namely AP. The content of AP was examined to be 98% by phenol-sulfuric acid colorimetric assay with SpectraMax M2^e^ microplate reader, as described by Rahman et al. [19].

### 2.3. Animals and Experimental Design

Seventy-two male ICR mice (18–21 g of weight) were purchased from Beijing Vital River Laboratory Animal Technology Co., Ltd. (Beijing, China) (Certificate SCXK-2016-0006). The mice were accommodated to a controlled environment at the ambient temperature of 18–22 °C and the humidity of 45–55% under a 12 h light/12 h dark cycle. The procedures of animals were strictly carried out in compliance with the Animal Ethics Committee of the Beijing Key Laboratory of Functional Food from Plant Resources and the guidelines for the care and use of laboratory animals of the National Institutes of Health.

**Figure 1:**
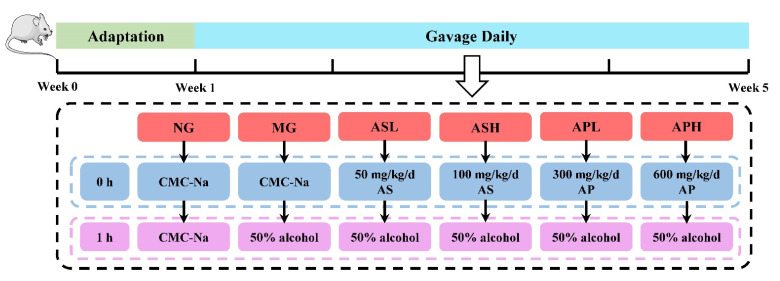
Schematic diagram of the whole animal experimental process.

After adaptation for 1 week with free access to feed and water, all mice were randomly divided into 6 groups (*n* = 12 per group): normal group (NG), model group (MG), Astragalus polysaccharides (low-dose group, APL; high-dose group, APH), Astragalus saponins (low-dose group, ASL; high-dose group, ASH). Mice in the NG and MG received 0.5% sodium carboxymethyl cellulose solution (CMC-Na) by oral gavage successively. After an interval of 1 h, the NG and MG were given orally 10 mL/kg body weight (bw) of CMC-Na and 50% alcohol, respectively. The APL and APH were supplied daily with 300 and 600 mg/kg bw of AP respectively, and the ASL and ASH were administrated with 50 and 100 mg/kg bw of AS by oral route, respectively. One hour later, the APL, APH, ASL, and ASH were given 50% alcohol. AP and AS were suspended in 0.5% CMC-Na. The volume of perfusion was altered via body weight of mice recorded every three days. The whole animal experiment lasted for four weeks. The schematic diagram of the whole animal experimental process is shown in Figure 1. At the end of the experiment, the mice were weighed and fasted for 12 h with water ad libitum. Blood samples were obtained from orbital venous plexus and placed at 4 °C for 12 h. Then the mice were sacrificed, and organ tissues (liver, kidney, spleen, and testis) and fat (abdomen and epididymis) were excised and weighed immediately for calculating the organ and fat indexes (organ index (%) = organ weight (g)/final body weight (g) × 100%; fat index (%) = fat weight (g)/final body weight (g) × 100%). The liver samples were divided into two parts, including immersion in 10% formalin solution for histopathology and homogenization, and kept at −80 °C for biochemical determination. Colonic contents were collected and stored at −80 °C for intestinal microbiota analysis.

### 2.4. Measurement of Biochemical Indicators in Serum and Liver

Blood samples were centrifuged at 4000× *g* for 15 min at 4 °C to collect serum. The levels of TG, TC, FFA, HDL-C, LDL-C, AST, ALT, ALP, and γ-GT in serum were detected by a Mindray BS-420 automatic biochemistry analyzer (Shenzhen Mindray Bio-Medical Electronics Co., Ltd., Shenzhen, China) according to the instructions of the corresponding kits.

Hepatic lipids were extracted from liver homogenates referring to the protocols of Zhao et al. [20]. The levels of TG, TC, and FFA in liver were determined using the corresponding detection kits. The MDA level and activities of SOD, CAT, GSH, and GSH-Px in liver were measured following the instructions of the corresponding assay kits. The contents of inflammatory cytokines (TNF-α, IL-6, and IL-1β) were detected using the corresponding ELISA kits. A BCA kit was used to determine the concentration of total protein of liver and the results were represented relative to the hepatic protein concentration.

### 2.5. Quantitative Real-Time PCR (qRT-PCR)

Total RNA was extracted from liver after grinding in 1 mL of TRIpure reagent. After assessing the concentration and purity by an ultraviolet–visible spectrophotometer (DS-11, Denovix, USA), the RNA was reverse transcribed into cDNA using a FastQuant RT Kit. The mRNA expression levels were determined by SuperReal PreMix Plus with SYBR Green according to the kit’s instruction. The primers used in the study are presented in the Table 1 and were synthesized by Sangon Biotech (Shanghai, China) Co., Ltd. (Shanghai, China). The gene GAPDH was regarded as an internal reference. The relative mRNA expression levels were calculated via the 2^−(ΔΔCt)^ method.

### 2.6. Histological Analysis

The liver tissues fixed in 10% formalin solution were embedded in paraffin and cut into slices. Then liver sections were stained with hematoxylin and eosin (H&E) and masson, respectively. The histological changes of liver sections were observed using a light microscope (BA-9000, Osaka, Japan).

### 2.7. Intestinal Microbiota Analysis

Gut microbiota analysis was performed by Majorbio Bio-Pharm Technology Co., Ltd. (Shanghai, China). Firstly, bacterial genomic DNA from colonic contents was extracted using a DNA extraction kit for soil and detected via a NanoDrop2000 spectrophotometer (Thermo Fisher Scientific, Waltham, MA, USA). The V3-V4 region of the 16S rDNA genes was amplified with universal primers (338F: 5′-ACTCCTACGGGAGGCAGCAG-3′; 806R: 5′-GGACTACHVGGGTWTCTAAT-3′) with barcodes. Then, the amplicons were confirmed through 2% agarose gel electrophoresis and purified by an AxyPrep DNA Gel Extraction Kit using the strips of 750 bp. Finally, the amplicon library was paired-end-sequenced on the Illumina Miseq platform (Illumina, San Diego, CA, USA).

### 2.8. Statistical Analysis

Data were presented via Mean ± standard deviation (SD). Significant differences between model and other groups were performed by independent-samples T-test using SPSS 25.0 (SPSS Inc., Chicago, USA). *p* < 0.05 and *p* < 0.01 were regarded as statistically and highly statistically significant, respectively.

## 3. Result

### 3.1. Effect of AP and AS on Food Intake, Body Weight, and Organ and Fat Index in ALD Mice

As displayed in Table 2, at the end of the experiment, the body weight and food intake of mice in the MG were obviously lower than those in the NG (*p* < 0.05). The index of liver, kidney, spleen, testis, and abdominal and epididymal fat in the MG were obviously higher than those in the NG (*p* < 0.05). Compared with the MG, only the low dose of AP could significantly reduce the liver index of alcohol-treated mice by 5.1% (*p* < 0.05). The kidney index in the APH and the ASH was clearly lower than those in the MG (*p* < 0.05). Interestingly, all treatment groups could obviously reduce the spleen index (vs. MG, *p* < 0.05) and only AP could reduce the testis index and the level of epididymal fat (vs. MG, *p* < 0.05).

### 3.2. Effect of AP and AS on Lipids of Serum and Liver in ALD Mice

**Figure 2:**
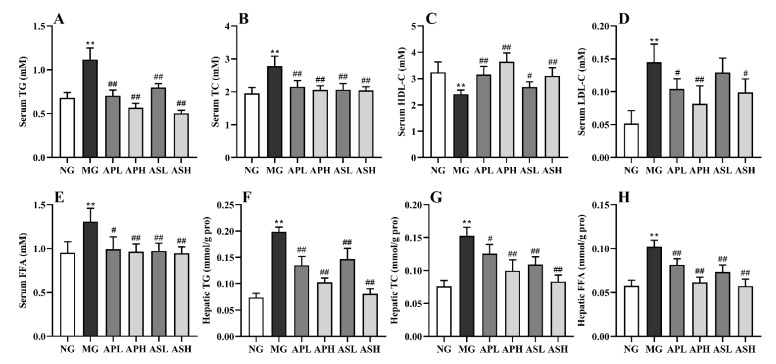
Effect of AP and AS on the serum and liver lipids in ALD mice. (**A**) Serum TG; (**B**) Serum TC; (**C**) Serum HDL-C; (**D**) Serum LDL-C; (**E**) Serum FFA; (**F**) Hepatic TG; (**G**) Hepatic TC; (**H**) Hepatic FFA. Data are expressed as mean ± SD (*n* = 12). ** *p* < 0.01, vs. NG; # *p* < 0.05, ## *p* < 0.01, vs. MG.

Figure 2 exhibits the influence of AP and AS on the serum lipids (TG, TC, HDL-C, LDL-C, and FFA) and hepatic lipids (TG, TC, and FFA). The contents of serum TG, TC, LDL-C, and FFA in the MG apparently plummeted, while the serum HDL-C level was significantly increased (vs. NG, Figure 2A–E, *p* < 0.01). Compared with the MG, the different dose of AP and AS had a varying degree of influence to mitigate the variation of the contents of serum TC, TG, FFA, HDL-C and LDL-C resulting from alcohol. Compared with the MG, the ASH had the best effect which reduced the serum TG by 55% (Figure 2A, *p* < 0.01). All active components could remarkably reduce the degree of TC by 22.66%–26.98% (vs. MG, Figure 2B, *p* < 0.01). The content of serum HDL-C in mice with components was markedly higher than that in the MG (Figure 2C, *p* < 0.01). Among them, the APH could increase the MG by 1.51-fold (vs. MG, Figure 2C). Figure 2D shows that all components markedly lowered the LDL-C level relative to the MG (*p* < 0.01). In the Figure 2E, all components reduced the serum FFA level compared to the MG (*p* < 0.05).

As shown in Figure 2F–H, the contents of hepatic TG and TC increased by 1.43- and 1.65-fold compared to the NG after alcohol consumption, respectively (*p* < 0.05). The AP and AS could decrease the content of hepatic TG in alcohol-treated mice (vs. MG, Figure 2F, *p* < 0.01). Similarly, the concentrations of hepatic TC in the APH, ASL, and ASH markedly declined (vs. MG, Figure 2G, *p* < 0.05), and the content of hepatic TC in the ASH was reduced to the equivalent level with the NG. In addition, AP and AS supplementation also drastically reduced the hepatic FFA level (Figure 2H, vs. MG, *p* < 0.01). The contents of hepatic FFA in the APH and ASH were almost the same as those in the NG.

### 3.3. Effect of AP and AS on the Hepatic Function in ALD Mice

**Figure 3:**
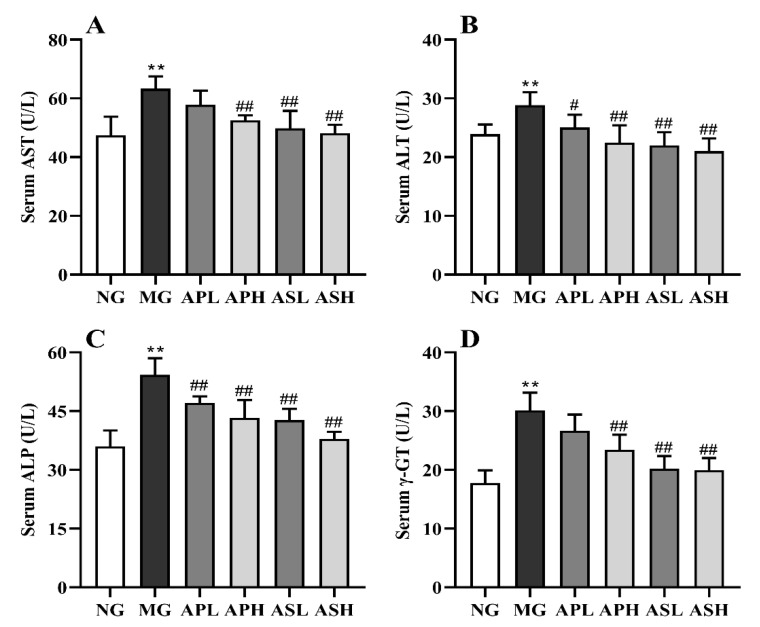
Effect of AP and AS on the hepatic function in ALD mice. (**A**) Serum AST; (**B**) Serum ALT; (**C**) Serum ALP; (**D**) Serum γ-GT. Data are expressed as mean ± SD (*n* = 12). ** *p* < 0.01, vs. NG; # *p* < 0.05, ## *p* < 0.01, vs. MG.

Figure 3 presents the variation of AST, ALT, ALP, and γ-GT in the serum of control and treated mice. The activities of these indexes showed a remarkable elevation after alcohol consumption (vs. NG, *p* < 0.01). The APH, ASL, and ASH significantly improved the content of AST (vs. MG, Figure 3, *p* < 0.01), and the improvement effect in the high-dose group was better than that in the low-dose group. The content of ALT in the APH, ASL, and ASH dropped sharply compared to the MG (Figure 3B, *p* < 0.01). In the Figure 3C, AP and AS significantly improved the level of ALP, and the ASH reduced the level of ALP by 35.68% (vs. MG, *p* < 0.01). Compared with MG, the APH, ASH, and ASL significantly reduced the concentration of γ-GT (*p* < 0.01). In terms of value, the ASH and ASL were even reduced by 33.78% compared to the MG.

### 3.4. Effect of AP and AS on the Liver Oxidative Stress in ALD Mice

**Figure 4:**
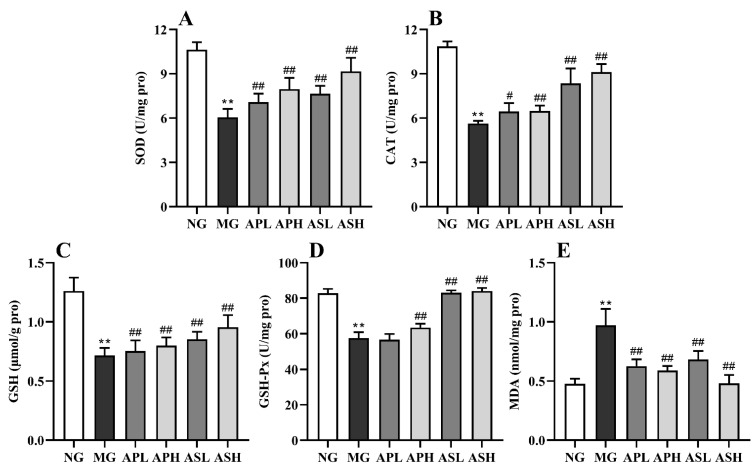
Effect of AP and AS on the antioxidant capacities in ALD mice. (**A**) SOD activity; (**B**) CAT activity; (**C**) GSH activity; (**D**) GSH-Px activity; (**E**) MDA level. Data are expressed as mean ± SD (*n* = 12). ** *p* < 0.01, vs. NG; # *p* < 0.05, ## *p* < 0.01, vs. MG.

Figure 4 shows that the level of hepatic MDA induced by alcohol was rapidly augmented, while the activities of CAT, SOD, GSH, and GSH-Px were apparently inhibited (vs. NG, *p* < 0.01). The AP and AS could reduce the production of lipid peroxidation products and oxidative stress caused by alcohol in varying degrees. These ingredients were more favorable for reducing the level of MDA, and the ASH was reduced 50% (vs. MG, Figure 4E, *p* < 0.01). The effect of the ASL and ASH on improving the activity of SOD was more obvious, and the effect of the ASH was also the best (vs. MG, *p* < 0.01). The ASL and ASH had the most obvious effect on the activities of CAT, GSH, and GSH-Px. Even after the ASH intervention, the GSH-Px value of ALD mice increased by 46.25%.

### 3.5. Effect of AP and AS on Inflammation Response in ALD Mice

**Figure 5:**
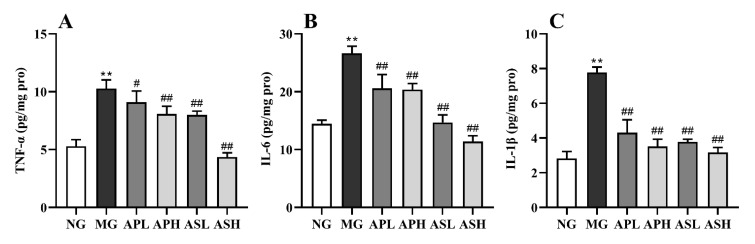
Effect of AP and AS on inflammation response in ALD mice. (**A**) Hepatic IL-1β; (**B**) Hepatic IL-6; (**C**) Hepatic TNG-α. Data are expressed as mean ± SD (*n* = 12). ** *p* < 0.01, vs. NG; # *p* < 0.05, ## *p* < 0.01, vs. MG.

Figure 5A–C shows the changes of pro-inflammatory factors in the livers of ALD mice to evaluate the effects of AP and AS on hepatic inflammatory stress. The level of pro-inflammatory cytokines in alcohol-treated mice was significantly enhanced compared to NG (*p* < 0.01). The value of IL-1β and IL-6 in mice administrated with AP and AS obviously declined (vs. MG, Figure 5A,B, *p* < 0.01). Compared with MG, the level TNF-α in the APH, ASL, and ASH dropped clearly (Figure 5C, *p* < 0.01). Interestingly, high doses of AS and AP were more effective than low doses. In addition, the levels of all the pro-inflammatory cytokines after ASH intervention were lower than half of those in the MG.

### 3.6. Effect of AP and AS on Oxidative Stress and Inflammation-Related Gene Expression in ALD Mice

**Figure 6:**
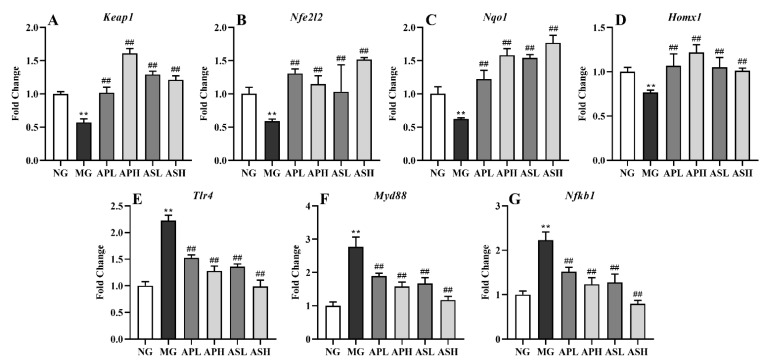
Effect of AP and AS on oxidative stress and inflammation-related gene expression in ALD mice. (**A**) *Keap1*; (**B**) *Nfe2l2*; (**C**) *Nqo1*; (**D**) *Homx1*; (**E**) *Tlr4*; (**F**) *Myd88*; (**G**) *Nfkb1*. Data are expressed as mean ± SD (*n* = 12). ** *p* < 0.01, vs. NG; ## *p* < 0.01, vs. MG.

According to Figure 6, the mRNA levels of *Keap1*, *Nfe2l2*, *Nqo1*, and *Hmox1* in the livers of alcohol-treated mice were significantly downregulated, while *Tlr4*, *Myd88,* and *Nfkb1* were remarkably upregulated (vs. NG, *p* < 0.01). AP and AS could significantly upregulate the mRNA expression of *Keap1*, *Nfe2l2*, *Nqo1*, and *Hmox1* and downregulate the mRNA expression of *Tlr4*, *Myd88,* and *Nfkb1* (vs. MG, *p* < 0.01). Taken together, AS showed more outstanding function on inhibiting inflammatory response than AP. The preventive effects of oxidant stress and inflammatory response in the high-dose groups were more beneficial than those in the corresponding low-dose groups.

### 3.7. Effect of AP and AS on Histopathological Variations of Livers in ALD Mice

**Figure 7:**
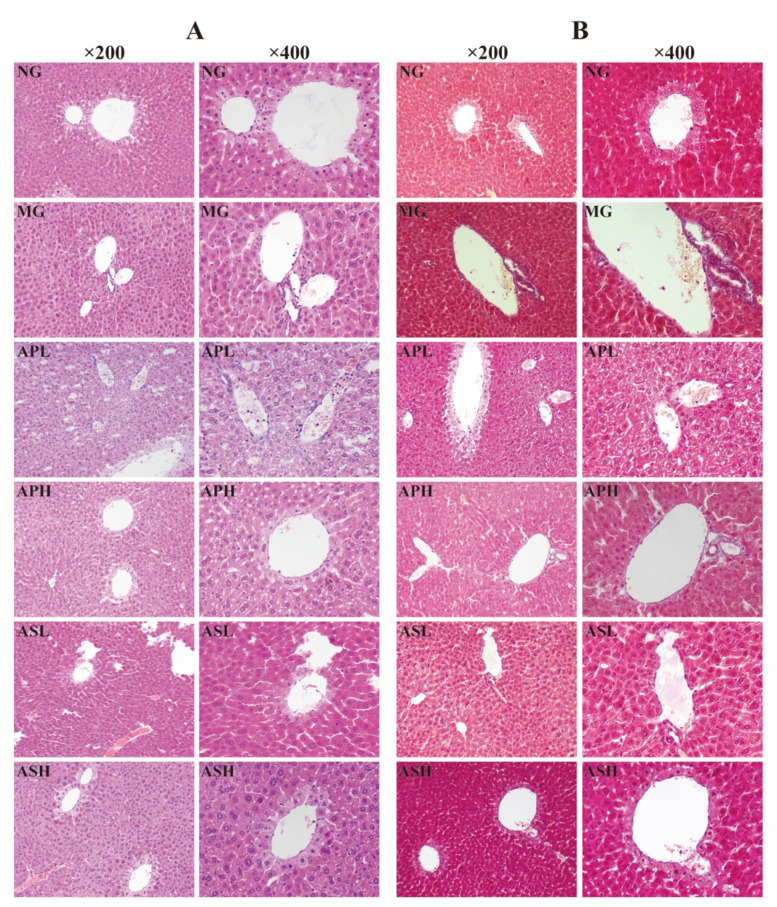
Histopathological detection of livers in ALD mice. (**A**) H&E staining in liver (×200 magnification and ×400 magnification); (**B**) Masson staining in liver (×200 magnification and ×400 magnification).

H&E and Masson staining were used to observe the pathological changes of liver tissues in ALD mice after AP and AS treatment. As shown in Figure 7A, the hepatocyte structure in the NG was normal, and there were no signs of steatosis, edema, and inflammatory cell infiltration. Differently, administration of alcohol for four weeks could lead to severe liver lesions with disordered liver cord, vesicular steatosis, and serious inflammatory cell infiltration. After the intervention of AS and AP, liver injury improved, and hepatocyte structure and liver cord were arranged neatly. There was mild steatosis and a small amount of inflammatory cell infiltration. In particular, there was no obvious inflammatory cell infiltration in the ASH.

Masson staining can be used to directly see whether the cells have fibrosis or not. As presented in Figure 7B, the hepatocytes in the NG did not undergo fibrosis. On the contrary, the liver of the MG is moderately fibrotic, accompanied by aggregation of collagen fibers. AS and AP can improve liver fibrosis caused by alcohol, and the fibrosis around the portal area and collagen fiber accumulation were reduced. Especially, the degree of hepatic fibrosis in the ASH was the lowest.

### 3.8. Effects of AP and AS on the Changes of the Colonic Microbiota Composition in ALD Mice

**Figure 8:**
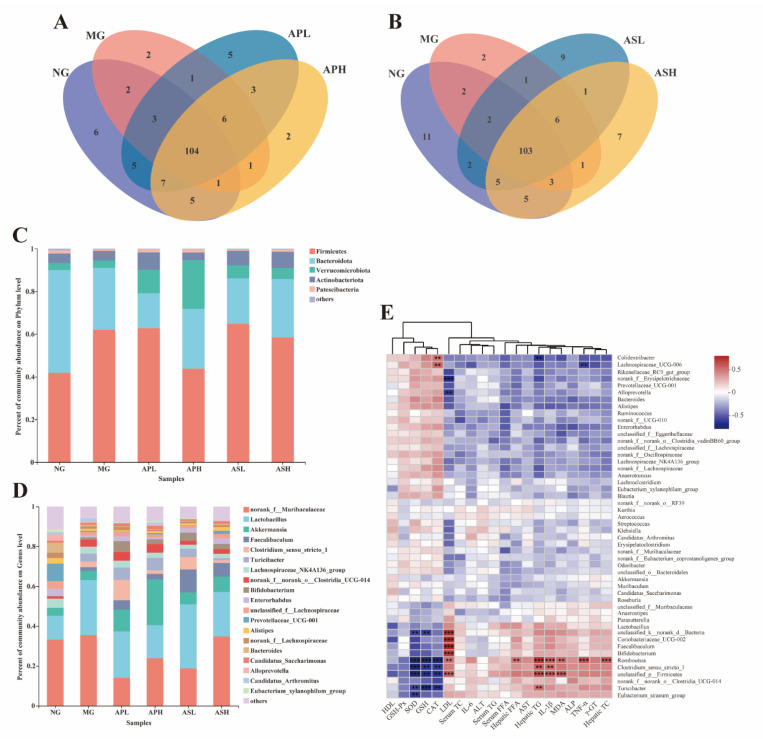
Effects of AP and AS on the changes of the colonic microbiota composition in ALD mice. (**A**) Venn diagrams of AP; (**B**) Venn diagrams of AS; (**C**) percent of community abundance at phylum level; (**D**) percent of community abundance at genus level; (**E**) heatmap of Spearman’s correlation analysis of the biological parameters and relative abundance of colonic microbiota at species level. *** *p* ≤ 0.001, ** *p* ≤ 0.01.

At the genus level, the Venn chart shows the number of OTUs and the amount of overlap in each group (Figure 8A,B). The results showed that the NG, MG, APL, and APH had a total of 104 OTUs (Figure 8A) and the NG, MG, ASL, and ASH had a total of 103 OTUs (Figure 8B). At the phylum level (Figure 8C), *unclassified_p_Firmicutes* and *Bacteroides* were the main bacteria in the NG flora (accounting for 82% of the total colony). Alcohol could cause the abundance of *unclassified_p_Firmicutes* to increase (29.08%) and the abundance of *Bacteroides* to decrease (61.99%). In the component groups, APH could reduce the *unclassified_p_Firmicutes* abundance value of ALD mice and slightly increase the *Bacteroides* abundance value. At the genus level (Figure 8D), the dominant bacteria in the NG were *norank_f_Muribaculaceae* (33.30%) and *Lactobacillus* (11.90%). The dominant bacteria in the intestinal microflora of ALD mice were *norank_f_Muribaculaceae* (35.50%) and *Lactobacillus* (27.64%). Under the intervention of AS and AP, the abundance of *norank_f_Muribaculaceae* and *Lactobacillus* in ALD mice decreased. Among them, the abundance of *norank_f_Muribaculaceae* in APL was only 14.06%, and the abundance of *Lactobacillus* in APH was 16.90%.

The correlation between microbial flora and biochemical indicators was analyzed by Spearman correlation heatmap (Figure 8E). The lipid parameters of liver and serum were mostly positively correlated with several types of bacteria, such as serum LDL-C showing a strong positive correlation with *Faecalibaculum*, *Coriobacteriaceae_UCG-002*, *unclassified_p_Firmicutes*, *Bifidobacterium*, *Coriobacteriaceae*, and *norank_d_Bacteria*. In addition, LDL-C was negatively correlated with *f_Erysipelotrichaceae*. Hepatic TG was significantly positively correlated with *Romboutsia* and *unclassified_p_Firmicutes*, and hepatic TC was only positively correlated with *Romboutsia*. Most serum oxidation markers were negatively correlated with some bacteria, such as SOD and CAT being negatively correlated with *Romboutsia*. In addition, the bacteria that were significantly negatively related to SOD were *unclassified_p_Firmicutes* and *Clostridium*. Similarly, GSH was negatively with *Turicibacter* and *Romboutsia*, but MDA was significantly positively correlated with *unclassified_p_Firmicutes*. In terms of inflammatory factors, IL-1β was positively correlated with *Firmicutes* and *Romboutsia*, and TNF-α was positively correlated with *Romboutsia*.

## 4. Discussion

ALD has become one of the leading causes of death among long-term drinkers [21,22]. Scholars believe that the progress of ALD is mainly related to oxidative stress, inflammatory cytokines, and apoptosis [23]. It is reported that AP and AS have antioxidant, anti-inflammatory, and anti-aging properties, which means they have the ability to improve the development of ALD [24,25]. In this study, AS and AP were successfully extracted, and the model of alcohol-induced liver injury in mice was established. Through the in vivo experiment, the improvement of AS and AP on alcohol-induced liver injury in mice was studied from the indexes of organ index, serum lipids, inflammatory factors, and oxidation markers. At the same time, the regulatory effects of AS and AP on intestinal microorganisms in mice with alcoholic liver injury were explored.

Except for the initial weight, all other indicators had significant differences between alcohol-treated mice and mice in the NG (Table 2). The results showed that alcohol intake can damage these organs to varying degrees, which is consistent with the results of our previous research [20]. AP could significantly improve the extent of alcohol damage to liver, kidney, spleen, and testicles (vs. MG). AS could obviously improve the index of liver and spleen and the fat of abdominal in alcohol-treated mice, and the high-dose AS had the best improvement on these indicators.

Long-term intake of alcohol will lead to excessive accumulation of TG, TC, and LDL-C in serum, and will produce a large number of free radicals which cause oxidative stress, resulting in the imbalance of blood lipid metabolism [26]. Hepatic TG, TC, and FFA are important indicators to judge dyslipidemia. The augment of FFA level will accelerate the synthesis of TG and TC in the liver [27]. In fact, current results show that all treatment groups could reduce serum and hepatic of TG, TC, and FFA levels (Figure 2A–H). Among the three indexes, ASH had the best improvement effect. Some studies showed that after lipid accumulation in the liver, the level of LDL-C was distinctly higher than those in NG, while the level of HDL-C was reversed [20]. In this study, AP and AS could reduce the content of serum LDL-C (vs. MG, Figure 2D). The content of HDL-C in all treatment groups show an upward trend (vs. MG, Figure 2C), which was consistent with Zhao et al. [28]. Similarly, all treatment groups could effectively reduce alcohol-induced fat deposition in mice (Figure 4D).

The ethanol intake could increase the AST and ALT activities, leading to the damage of hepatocytes. Therefore, the level of serum AST and ALT could reflect the degree of liver damage, to a certain extent [29,30]. ALP is mainly distributed in the bile capillaries of stem cells, and γ-GT is mainly distributed in the hepatocyte membrane. Their levels can be used in the diagnosis of cholestatic liver disease or obstructive liver disease [31,32]. This study shows that AP and AS could obviously improve the above four liver function indexes, and ASH had the best improvement effect (vs. MG, Figure 3). Particularly, the levels of ALT and ALP in the high dose of saponins group were close to that of NG (Figure 3B,C). Therefore, AS had the best effect on improving liver function in a dose-dependent manner.

Oxidative stress plays an important role in alcoholic liver injury. SOD, CAT, and GSH can scavenge excessive the production of free radicals in the body and achieve the effect of antioxidation. At the same time, MDA, as a peroxidation product, can be used to reflect the degree of oxidative stress [33,34]. Some studies showed that intake of alcohol can increase the content of MDA and decrease the content of CAT, SOD, GSH, and GSH-Px in the liver [35]. In this study, ASH and ASL could significantly increase the content of CAT, SOD, GSH, and GSH-Px in the liver of alcohol-treated mice, and the improvement effect was the best among all the treatment groups (Figure 4), although all the treatment groups could reduce the content of MDA in the liver of alcohol-treated mice. Therefore, both AS and AP could improve alcohol-induced oxidative stress in the liver of mice to some extent. Kelch-like ECH-associated protein 1 (KEAP1)/NF-E2-related factor 2 (NRF2) signaling pathway is a classic antioxidant pathway in hepatocytes which reduces the level of oxidative stress and regulates the balance of the body system [36]. During oxidative stress, KEAP1 (coded by *Keap1*) and NRF2 (coded by *Nfe2l2*) are separated into the nucleus and bind to the ARE site in the nucleus, activating the gene expression of downstream Heme Oxygenase 1 (HO-1, coded by *Hmox1*) and Homo sapiens NADPH quinone dehydrogenase 1 (NQO1, coded by *Nqo1*), promoting the release of antioxidant enzymes and regulating the level of oxidative stress [37]. In this experiment, compared with MG, AP and AS could significantly upregulate the expression of *Keap1*/*Nfe212*/*Nqo1*, indicating that AP and AS could inhibit oxidative stress resulting from the KEAP1/NRF2 pathway.

There is a close relationship between liver inflammation and oxidative stress. Due to the production of a large number of reactive oxygen free radicals, oxidative stress will promote the release of inflammatory factors, such as IL-1β, IL-6 and TNF-α, which leads to liver inflammation [38]. In particular, it could reduce the contents of IL-6 and TNF-α in the liver to the level of the NG, and there was a significant dose relationship between high and low doses (Figure 5B,C). TLR4/MyD88/NF-κB is a classic inflammatory signaling pathway that induces alcoholic liver injury in mice [39,40]. Tlr4 can be activated after injury, and it can promote the expression of inflammatory factor IL-6 by promoting the expression of downstream factors MyD88 and NF-κB [41]. In this study, AP and AS could downregulate the gene expression levels of TLR4 (coded by *Tlr4*), MyD88 (coded by *Myd88*), and NF-κB (coded by *Nfkb1*), demonstrating that AP and AS could suppress inflammation through the TLR4/MyD88/NF-κB pathway. Similarly, from this histological observation experiment, it was found that each treatment group could effectively reduce the degree of liver inflammation and fibrosis (Figure 6).

Many studies have shown that intestinal flora plays an important role in the development of various liver diseases [42]. Alcohol can destroy the intestinal microflora system, which in turn increases the pro-inflammatory environment of the liver [43]. Clinical trials have showed that the abundance of the intestinal microflora of ALD patients will change [44]. In this experiment, our results showed that in the intestinal flora of alcohol-treated mice, the abundance of harmful bacteria (*norank_f_Muribaculaceae* and *unclassified_p_Firmicutes*) increased and the abundance of beneficial bacteria (*Akkermansia*) decreased, which were consistent with the results of previous studies [45,46]. After treatment with a high dose of AP, the proportion of gut microbiota in ALD mice significantly improved, and the abundance of harmful bacteria decreased. Furthermore, the abundance of *norank_f_Muribaculaceae* and *Lactobacillus* at the genus level showed an obvious reduction, and *Lactobacillus* was a kind of beneficial bacteria that could regulate inflammation [47]. The abundance of *Lactobacillus* in the ASL was higher than that in MG. These results indicate that AS and AP could improve the intestinal microflora imbalance caused by alcohol.

## 5. Conclusions

To sum up, the alleviating effects of AS and AP on alcohol-induced liver damage were attributed to suppressing the lipid accumulation in serum and liver, the levels of inflammatory cytokines and hepatic fibrosis, and enhancing the liver function and the activities of oxidant status markers, as well as regulating the gut microbiota disorder. In addition, the improving effect of AS on preventing ALD was more efficient than that of AP. The high dose of AS or AP showed more beneficial function than the low dose of AS or AP. Moreover, qRT-PCR analysis demonstrated that AS and AP could attenuate oxidative stress and inflammatory response induced by alcohol through KEAP1/NRF2 and TLR4/MyD88/NF-κB signaling pathway, respectively. Those results provide new insights for the application of Astragalus with managing ALD.

## Figures and Tables

**Table 1:** Primer sequences of genes used for qRT-PCR.

Gene	Forward Primer (5′-3′)	Reverse Primer (5′-3′)
*Gadph*	TCTCCTGCGACTTCAACA	TGTAGCCGTATTCATTGTCA
*Keap1*	CAGATTGACAGCGTGGTT	GCAGTGTGACAGGTTGAA
*Nfe2l2*	GTGCTCCTATGCGTGAAT	TCTTACCTCTCCTGCGTATA
*Hmox1*	AGGTCCTGAAGAAGATTGC	TCTCCAGAGTGTTCATTCG
*Nqo1*	ATGAAGGAGGCTGCTGTA	AGATGACTCGGAAGGATACT
*Tlr4*	TGACATTCCTTCTTCAACCA	CACAGCCACCAGATTCTC
*Myd88*	CCGTGAGGATATACTGAAGG	TTAAGCCGATAGTCTGTCTG
*Nfkb1*	AGACAAGCAGCAGGACAT	CCAGCAACATCTTCACATC
*Gadph*	TCTCCTGCGACTTCAACA	TGTAGCCGTATTCATTGTCA

**Table 2:** Effects of AP and AS on body weight, food intake, and organ and fat index of mice ^1^.

Groups	NG	MG	APL	APH	ASL	ASH
Initial weight (g)	28.71 ± 1.46	28.47 ± 1.97	28.77 ± 1.32	28.71 ± 1.37	28.84 ± 1.48	28.66 ± 1.51
Final weight (g)	34.17 ± 3.11	29.68 ± 2.36 **	29.14 ± 1.50	30.05 ± 2.69	30.01 ± 1.39	29.50 ± 3.07
Food intake (g/d)	4.90 ± 0.46	3.63 ± 0.66 **	3.42 ± 0.70	3.53 ± 0.65	3.30 ± 0.75	3.73 ± 0.70
Liver index (%)	3.69 ± 0.36	3.90 ± 0.25 **	3.71 ± 0.26 #	3.89 ± 0.15	4.10 ± 0.37	3.89 ± 0.19
Kidney index (%)	1.35 ± 0.12	1.46 ± 0.10 *	1.44 ± 0.08	1.34 ± 0.10 #	1.36 ± 0.08	1.34 ± 0.04 #
Spleen index (%)	0.27 ± 0.04	0.31 ± 0.03 *	0.27 ± 0.02 #	0.26 ± 0.03 #	0.27 ± 0.02 #	0.26 ± 0.03 #
Testis index (%)	0.70 ± 0.10	0.82 ± 0.07 *	0.71 ± 0.04 #	0.71 ± 0.07 #	0.81 ± 0.06	0.85 ± 0.02
Abdominal fat (%)	0.24 ± 0.04	0.36 ± 0.08 **	0.26 ± 0.14	0.29 ± 0.17	0.32 ± 0.08	0.24 ± 0.07 #
Epididymal fat (%)	1.13 ± 0.20	1.51 ± 0.27 **	1.20 ± 0.29 #	1.16 ± 0.32 #	1.39 ± 0.15	1.48 ± 0.27

^1^ Data are expressed as mean ± SD (*n* = 12). * *p* < 0.05, ** *p* < 0.01, vs. NG; ^#^ *p* < 0.05, vs. MG.
